# LncRNAs regulate the cyclic growth and development of hair follicles in Dorper sheep

**DOI:** 10.3389/fvets.2023.1186294

**Published:** 2023-07-31

**Authors:** Haoran Sun, Ke Meng, Yifan Wang, Yayan Wang, Xiaochun Yuan, Xinhai Li

**Affiliations:** College of Animal Science and Technology, Ningxia University, Yinchuan, China

**Keywords:** sheep, hair follicle, lncRNA, anagen, telogen, RNA-seq

## Abstract

**Introduction:**

Hair follicles in Dorper sheep are characterized by seasonal cyclic growth and development, consequently resulting in hair shedding during spring. The cyclic growth and development of hair follicles are regulated by several influencing factors such as photoperiods, hormones, age of the animal, genes, long non-coding RNAs (lncRNAs), and signaling pathways.

**Methods:**

In the present study, skin samples of five shedding sheep (S), used as experimental animals, and three non-shedding sheep (N), used as controls, were collected at three time points (September 27, 2019; January 3, 2020; and March 17, 2020) for RNA sequencing (RNA-seq) technology. Nine different groups (S1-vs-S2, S1-vs-S3, S2-vs-S3, N1- vs-N2, N1-vs-N3, N2-vs-N3, S1-vs-N1, S2-vs-N2, and S3-vs-N3) were compared using FDR < 0.05 and log 21 FC >as thresholds to assess the differences in the expression of lncRNAs.

**Results and discussion:**

In total, 395 differentially expressed (DE) lncRNAs were screened. Cluster heatmap analysis identified two types of expression patterns, namely, high expression during the anagen phase (A pattern) and high expression during the telogen phase (T pattern). Gene Ontology (GO) enrichment analysis and Kyoto Encyclopedia of Genes and Genomes (KEGG) enrichment analyses revealed that the target genes were largely enriched in the Estrogen signaling pathway, PI3K-Akt signaling pathway, Fc gamma R-mediated phagocytosis, and cell adhesion molecules (CAMs), which are associated with hair follicle cyclic growth and development-related pathways. In addition, 17 pairs of lncRNAs-target genes related to hair follicle cyclic growth and development were screened, and a regulatory network was constructed. Altogether, candidate lncRNAs and their regulated target genes were screened that contributed to sheep hair follicle cyclic growth and development. We believe these findings will provide useful insights into the underlying regulatory mechanisms.

## 1. Introduction

The hair follicles of certain animal species, such as Inner Mongolian cashmere goats and Dorper sheep, are characterized by seasonal cyclic growth and development. This process results in hair shedding during spring. The cyclic growth and development of hair follicles occur in three phases, namely, anagen, catagen, and telogen (1). Anagen is the most active phase of hair follicle growth, during which hairs grow rapidly and form a complete hair shaft (HS) (2), and the duration of anagen determines the length of the hair. During the catagen phase, HS stops growing; cell proliferation and differentiation capacity begin to decline; cells start undergoing apoptosis; and hair follicles rapidly degenerate (3). Subsequently, the telogen phase begins, during which the biological activity of hair follicles is the weakest and HS decreases (3). During the cyclic growth and development of hair follicles, a large and complex regulatory network comprising genes, microRNAs (miRNAs), long non-coding RNAs (lncRNAs), and other regulators is formed through mutual inhibition or synergistic effects (3).

Several pathways, such as WNT (4), MAPK (5), BMP (6), and TGF-β (7) signaling pathways, have been implicated in the regulation of hair follicle growth and development. Any abnormality in the ligands, receptors, and signal transduction molecules of these signaling pathways can affect the development of hair follicles in animals and changes in hair growth (3). In these pathways, *BMP* [*BMP4* (8), *BMP7* (9)], *FGFs* [*FGF20* (10), *FGF10* (11), *FGF4* (12)], *Sox2* (13), *Sox18* (14) are intricately associated with hair follicle development and wool bending. *NF-κB* (15), *WNT10b* (16), *LEF1*, *IGF1R* (17), and *Noggin* (18) have been reported to promote early hair follicle morphogenesis. Similarly, *DKK1* (16), *PF4* (19), *FGF5*, and *FGF18* (20) have been found to inhibit the growth of hair follicles. Numerous studies have demonstrated the role of lncRNAs in the complex regulatory network. For instance, Yue et al. determined the functions of lncRNAs and mRNAs in sheep skin by strand-specific RNA sequencing (ssRNA-seq) during the initiation phase of secondary hair follicle (SHF) development. They concluded that crucial differentially expressed genes (DEGs) and lncRNAs influence hair follicle initiation (21). Yang et al. screened lambda cyclic growth-specific lncRNAs and found that 6,127 lncRNAs were expressed during anagen and telogen phases, of which 54 were significantly differentially expressed (32 up-regulated and 22 down-regulated) (22). LncRNA15479 was identified as a crucial molecule that was intricately associated with the anagen phase of hair follicles and involved in the formation of keratin by target knockdown experiments (22). In addition, *PlncRNA-1* was found to be responsible for the biological changes in the Wnt/β-catenin signaling pathway by regulating *TGF-β1*, thereby participating in the biological regulation of hair follicle stem cell proliferation and differentiation (23). In the present study, we screened and identified lncRNAs related to hair follicle cyclic growth and development. However, the regulatory mechanisms warrant further investigation.

Shedding sheep (S) with cyclic hair follicle growth and development were selected as experimental animals and non-shedding (N) sheep as the control. The expression of lncRNAs was determined at three different time points by RNA sequencing technology (RNA-seq), followed by the screening of lncRNAs by differential expression analysis and expression pattern analysis. Finally, candidate lncRNAs were screened by lncRNAs-target genes synergy analysis. The specific analysis flow of lncRNA is shown in Supplementary Figure 1. We investigated the lncRNAs affecting anagen and telogen phases of sheep hair follicles and explored the specific regulatory mechanisms to provide a theoretical basis for breeding automatic shedding meat sheep.

## 2. Materials and methods

### 2.1. Experimental animals and sample collection

The study was approved by the Experimental Animal Welfare and Ethics Committee of Ningxia University (Animal Ethics approval no. NXU-19-018). The experimental sheep were obtained from the sheep farm of Ningxia China Animal Husbandry Yilin Livestock Co., Ltd. The skin samples were collected from five 2-year-old shedding Dorper ewes and three 2-year-old non-shedding Dorper ewes on September 27, 2019; January 3, 2020; and March 17, 2020, from the side of the sheep’s body (the junction of the posterior edge of the last rib and the midline of the body). The sample size was approximately 2 cm^2^ of skin tissue. Samples were split and stored in liquid nitrogen. The hair follicles were in the anagen phase on September 27, 2019, for both shedding and non-shedding sheep. The hair follicles were in the telogen phase on January 3, 2020, for shedding sheep, whereas the hair follicles of non-shedding sheep were still in the anagen phase. The hair follicles were in the early anagen phase on March 17, 2020, for shedding sheep, whereas those of non-shedding sheep were in the slow anagen phase. The samples were divided and stored in liquid nitrogen.

### 2.2. RNA isolation, library preparation, and sequencing

First, 50–100 mg of sheep skin samples were obtained from eight Dorper sheep separately and placed in mortars. They were ground into powder using a pestle under liquid nitrogen. Next, the powder was put in a 1.5 mL centrifuge tube and 1 mL of the TRIzol reagent (Invitrogen, USA) was added. It was kept at room temperature for 5 min. Next, 0.2 mL chloroform (Tianjin Regent Chemical Co., Ltd) was added, shaken for 15 s, and allowed to stand for 10 min. Next, the supernatant was centrifuged at 4°C using a benchtop high-speed refrigerated centrifuge (Kate’s Laboratory Instruments Co., Ltd., TGL 18 M) at 12,000 *g* for 15 min, following which the supernatant was placed in another centrifuge tube. Third, 0.5 mL isopropanol (Tianjin reagent Chemical Co., Ltd.) was added to the supernatant, the liquid was mixed in the tube gently, and it was made to stand for 10 min at room temperature and centrifuged (using the method above). After centrifugation, the supernatant was discarded and the precipitate was retained. Finally, 1 mL of 75% ethanol (Yantai Shuang Chemical Co., Ltd.) was added to the precipitate, which was gently obtained. It was centrifuged at 4°C at 7500 *g* for 10 min and the supernatant was discarded. It was air dried (15 min) and an appropriate volume of RNase-free water (Tiangen Biochemical Technology; Beijing) was added to dissolve the precipitate (65°C for 10–15 min). The purity and concentration of the extracted RNA were measured using the NanoDrop 2000 spectrophotometer (NanoDrop 2000, UV-Vis spectrophotometer, USA) and Agilent 2100 Bioanalyzer RNA Nano 6000 kit of the Agilent Bioanalyzer 2100 system (Agilent Technologies, CA, USA). The extracted total RNA was reverse transcribed into cDNA using a reverse transcription kit (Nanjing Novozymes Biotechnology Co., Ltd.). The library was constructed using the illumina Ribo-Zero Gold (Human/Mouse/Rat) Kit (MRZG12324, illumine) and NEB#7490 Kit (NEB E7490L, New England Biolabs) according to the manufacturer’s instructions. Subsequently, 24 cDNA libraries from eight sheep at three different time points were generated. The quality of the constructed library was evaluated using Agilent 2100, and after passing library inspection, 24 cDNA libraries were sequenced together using the Illumina HiSeqTM 4000 (Illumina; San Diego, CA, USA).

### 2.3. Quality control

The quality of the raw data was assessed using fastp (24). The procedure included the following steps: (1) removing reads containing adapters, (2) removing reads with a percentage of unknown bases (N) greater than 10%, (3) removing reads consisting entirely of A bases, and (4) removing low-quality reads (reads with Q phred ≤  20 bases accounting for more than 50% of the entire read length). The remaining clean reads were mapped to the reference genome Oar_rambouillet_v1.0 using HISAT2 (25).

### 2.4. Comprehensive analysis of lncRNAs

#### 2.4.1. Identification of lncRNAs

The transcripts of each biological sample were reconstructed using the merge function of StringTie (26), and the obtained transcripts were compared with known transcripts from the genome annotation to exclude known transcripts. The coding ability of the new transcripts was then performed using both CPC2 (27) and CNCI (28) software, and the intersection of transcripts without coding potential was taken as a reliable prediction, and the existing lncRNA annotations of the reference genome were excluded. Rfam is a database containing information on various lncRNA families, including conserved regions of RNA secondary structure, mRNA cis-acting elements, and other RNA elements. It classifies ncRNAs into different families based on their common ancestry at the evolutionary level, and each family consists of secondary structures and covariates predicted from multiple sequence comparisons. To better annotate lncRNA at the level of evolution, we used Infernal (v1.1.2) (29) to classify all predicted lncRNAs according to their conserved sequence and secondary structure through multiple sequence alignment, secondary structure, and a covariance model. In addition, we used Cuffcompare (30) to compare the positions of lncRNAs with the positions of protein-coding RNAs. Based on this lncRNA were then classified into intergenic lncRNA (cuffcompare class code u,), intronic lncRNA (cuffcompare class code i), sense lncRNA (cuffcompare class code j, o), antisense lncRNA (cuffcompare class code x) and other lncRNA (remaining cuffcompare class codes).

#### 2.4.2. Analysis of differentially expressed lncRNAs

The expression of lncRNAs was displayed as raw read count and FPKM (Fragments Per Kilobase of transcript per Million mapped reads) value. Many factors influence raw reads such as transcript length and the total number of reads. Raw reads were not conducive to the comparison of differential lncRNAs between the samples. To ensure the accuracy of the subsequent analysis, we first corrected the sequencing depth and subsequently, the length of the transcript to obtain the FPKM value of lncRNA before further analyses. The transcripts were quantified with RSEM (31). Based on the lncRNA expression information, we conducted principal component analysis (PCA) using R^1^, and calculate the Pearson correlation coefficient (cor) between samples. The more similar the samples were, the closer they were reflected in the PCA plot, and the samples from different effective treatments tended to show their own aggregated distribution. Differential expression analysis of lncRNAs was performed using DESeq2 (32), which provided statistical analyses using a model based on the negative binomial distribution. Nine different groups (S1-vs-S2, S1-vs-S3, S2-vs-S3, N1-vs-N2, N1-vs-N3, N2-vs-N3, S1-vs-N1, S2-vs-N2, S3-vs-N3) were compared using FDR < 0.05 and |log2FC|>1 as thresholds to assess the statistical significant differences in the expression of lncRNAs. The differentially expressed (DE) lncRNAs between the final different comparison groups were considered a concurrent set.

#### 2.4.3. Cluster heatmap analysis and expression pattern analysis of differentially expressed lncRNAs

A cluster heatmap analysis was conducted using the FPKM values of DE lncRNAs for three phases of two groups to explore the expression pattern of lncRNAs using the OmicShare tools^2^, a free online platform for data analysis. Whether the lncRNAs have similar expression patterns were determined using the high and low expression (FPKM) and trend directions of lncRNAs at three different time points in the S and N groups. For example, both lncRNA 1 and lncRNA 2 displayed a high-low-high expression trend in the S group, and both exhibited an all-time elevated expression trend in the N group, which we considered as similar expression patterns. LncRNAs with similar expression patterns often have similar functions or are involved in the same metabolic processes or pathways.

### 2.5. Target gene prediction and correlation analysis of differentially expressed lncRNAs

#### 2.5.1. Target genes prediction of differentially expressed lncRNAs

To explore the function of lncRNAs, we predicted cis-target genes (neighboring genes), trans-target genes (co-expressed genes), and antisense lncRNA genes of identified lncRNAs using mRNAs sequence and expression information from mRNA sequencing data (33) of the same experimental sheep as in this study. Trans-acting lncRNAs influenced the gene expression in diverse biological processes at the transcriptional or post-transcriptional level, whereas cis-acting lncRNAs epigenetically regulated the nearby genes only at the transcriptional level (34). LncRNAs can trans-regulate distant target genes because they have the same expression pattern (35). Therefore, genes with |cor| ≥ 0.95 with this lncRNA were selected as trans-target genes. A cis-acting lncRNA is defined as the one regulating protein-coding genes within the proximity of its genomic locus (10 kb in our case) (36). Subsequently, the cor between lncRNA expression and cis-target genes expression was calculated, and |cor| ≥ 0.8 was retained. Antisense lncRNAs are transcription products from the opposite strand of the protein-coding or sense strand, which regulate the corresponding gene by gene silencing or by degrading sense transcripts (37, 38). The software RNA-plex (39) was used to predict the complementary correlation of antisense lncRNAs and target genes (antisense-role). Subsequently, the cor between lncRNA and gene expression was calculated, and |cor| ≥ 0.8 was retained.

#### 2.5.2. Differentially expressed analysis and expression pattern analysis of target genes

For the predicted results of target genes, DE genes with FDR < 0.05 and |log2FC|>1 were screened according to the different comparison groups (S1-vs-S2, S1-vs-S3, S2-vs-S3, N1-vs-N2, N1-vs-N3, N2-vs-N3, S1-vs-N1, S2-vs-N2, S3-vs-N3) of this study using the DESeq2 software based on the pre-mRNA sequencing data (33). Afterward, their expression patterns were mapped using the OmicShare tool (see footnote 2).

#### 2.5.3. GO and KEGG enrichment analysis of target genes and construction of the regulatory network

Gene Ontology (GO) enrichment analysis of target genes after target gene prediction and screening were performed using the GOseq R software package. Signaling pathway enrichment analysis was performed using the Kyoto Encyclopedia of Gene and Genomes (KEGG) database. GO terms and pathways with *q*-value (adjusted *p*-value) < 0.05 were considered significantly enriched. Candidate lncRNAs-target genes-pathways were screened and the regulatory network was visualized using Cytoscape (v3.9.1) (40).

### 2.6. qRT-PCR

To validate the accuracy of the RNA-seq results, six DE lncRNAs were selected for quantitative real-time polymerase chain reaction (qRT-PCR). Glyceraldehyde 3-phosphate dehydrogenase (*GAPDH*) was used as the reference gene for analysis. The qRT-PCR primers were designed using the Primer Premier 5.0 software (41), and primer information is shown in Table 1. The 2^−ΔΔCt^ method was used to process the qRT-PCR results (42).

**Table 1:** Information of fluorescent quantitative primers.

Type	Gene/LncRNA ID	Primer sequences (5′ → 3′)	Product size (bp)
Reference gene	GAPDH	F: TCCACGGCACAGTCAAGG	239
		R: CACGCCCATCACAAACAT	
	MSTRG.22781.1	F: AGTGAGTGAGGAGGTAAGGTGC	192
		R: GAGAGAGTGAAGGGGAGGAGAG	
	MSTRG.13836.1	F: CTTCTCCCGCCTTCAATCTTT	281
		R: AGTGGGTTGCCATCTTCTTCC	
	MSTRG.23001.1	F: CAGATCCAGGCGAAGGGAGG	238
LncRNA		R: GATGCGGGGCTGAGGAGAGT	
	XR_001024822.3	F: TGGAGACCACCAAAGGAAAT	202
		R: GTCAATAAGGCTACACACGG	
	MSTRG.26640.2	F: GTGACTGTCCTCGCCGTGCC	214
		R: ACCCAACCCCTGCCTTCCTC	
	MSTRG.1595.1	F: GGAGGCTATGGATGTGGCT	121
		R: CTTCAGATGTTGGAGTGGG	

## 3. Results

### 3.1. Overview of RNA-seq

**Figure 1:**
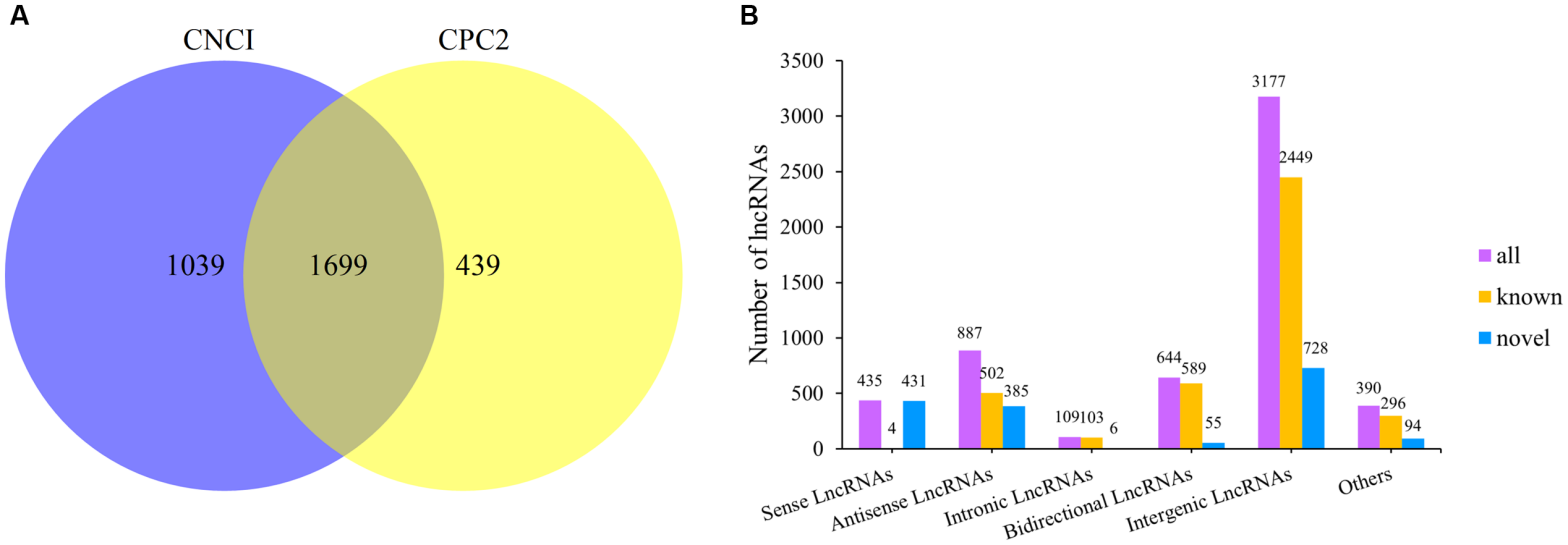
Classification of lncRNAs and prediction of novel lncRNAs. **(A)** Venn diagram of novel lncRNAs prediction. **(B)** LncRNA type statistics.

After extracting total RNA from eight Dorper sheep during three periods, we constructed 24 transcriptome libraries from skin samples and sequenced them individually. A total of 2,184,682,876 raw reads were produced using the Illumina HiSeqTM 4000. After trimming to remove adaptor sequences, and discarding low-quality sequences, 2,178,092,054 clean reads were retained (accounting for 99.69% of raw reads) (Table 2). The percentage of Q20 of each sample was not less than 97.28% and the percentage of the Q30 base of each sample was not less than 92.75%. The GC content ranged between 48.46 and 53.21%. The average GC content value with a standard deviation was 1.16%. Subsequently, we mapped the clean reads to the reference genome Oar_rambouillet_v1.0 using HISAT2. The matching rate was above 93.65% (Table 2). Quality control results confirmed the reliability of sequencing results and adequacy for further data processing. We reconstructed 9,939 transcripts and 6,970 genes using StringTie. After coding potential analysis using the software CNCI and CPC2, 1,699 novel lncRNA transcripts were identified (Figure 1A). Five different categories of lncRNAs were classified based on their positions relative to the protein-coding genes, including intergenic lncRNAs, bidirectional lncRNAs, intronic lncRNAs, antisense lncRNAs, and sense lncRNAs (Figure 1B).

**Table 2:** RNA-seq quality control result.

Sample	Raw datas	Clean datas (%)	GC content (%)	N (%)	Q20 value (%)	Q30 value (%)	Unique mapped reads (%)	Mapping ratio (%)
S1-1	80,305,380	80,151,468 (99.81%)	48.46%	0	97.46%	93.03%	85.82%	93.65%
S1-2	88,681,572	88,514,636 (99.81%)	50.29%	0	97.71%	93.65%	84.78%	96.29%
S1-3	88,639,604	88,435,920 (99.77%)	50.42%	0	97.86%	93.92%	84.18%	95.94%
S1-4	82,302,900	82,082,050 (99.73%)	52.42%	0	97.80%	93.95%	80.81%	96.52%
S1-5	95,132,446	94,861,532 (99.72%)	50.09%	0	97.83%	93.70%	83.97%	95.97%
N1-1	102,715,284	102,451,238 (99.74%)	48.74%	0	97.94%	93.97%	86.75%	96.57%
N1-2	125,141,914	124,812,746 (99.74%)	50.20%	0	98.00%	94.16%	82.65%	96.40%
N1-3	89,039,214	88,758,724 (99.68%)	49.64%	0	97.53%	93.04%	85.44%	96.43%
S2-1	101,606,896	101,305,036 (99.70%)	48.95%	0	97.60%	93.25%	87.06%	95.82%
S2-2	111,482,124	111,138,096 (99.69%)	49.97%	0	97.78%	93.72%	85.72%	96.13%
S2-3	78,132,386	77,918,790 (99.73%)	49.69%	0	98.02%	94.30%	86.16%	96.70%
S2-4	88,934,292	88,662,238 (99.69%)	51.30%	0	97.70%	93.69%	81.77%	94.38%
S2-5	88,841,684	88,527,154 (99.65%)	50.29%	0	97.75%	93.79%	85.05%	95.57%
N2-1	83,172,554	82,959,962 (99.74%)	49.99%	0	98.00%	94.24%	85.03%	96.95%
N2-2	93,246,142	92,973,282 (99.71%)	51.48%	0	98.02%	94.44%	81.72%	94.47%
N2-3	90,875,762	90,626,904 (99.73%)	51.19%	0	97.96%	94.14%	84.04%	96.97%
S3-1	94,155,288	93,804,848 (99.63%)	51.32%	0	97.28%	92.75%	83.88%	95.15%
S3-2	100,794,688	100,434,556 (99.64%)	50.61%	0	97.69%	93.75%	82.96%	94.34%
S3-3	79,139,424	78,899,488 (99.70%)	51.62%	0	97.97%	94.34%	80.02%	95.91%
S3-4	79,187,140	78,889,182 (99.62%)	53.21%	0	97.64%	93.82%	82.35%	94.68%
S3-5	80,678,998	80,361,468 (99.61%)	51.71%	0	97.82%	94.05%	77.34%	92.41%
N3-1	80,882,400	80,589,776 (99.64%)	50.98%	0	97.92%	94.33%	82.01%	95.75%
N3-2	95,377,002	95,067,686 (99.68%)	50.02%	0	97.61%	93.40%	84.99%	95.83%
N3-3	86,217,782	85,865,274 (99.59%)	52.11%	0	97.64%	93.68%	78.93%	94.80%

For example, S1-1, S represent shedding group, the first 1 is the first stage of S, the second 1 is the first sheep, and so on.

### 3.2. Correlation analysis of lncRNAs

#### 3.2.1. Principal component analysis

**Figure 2:**
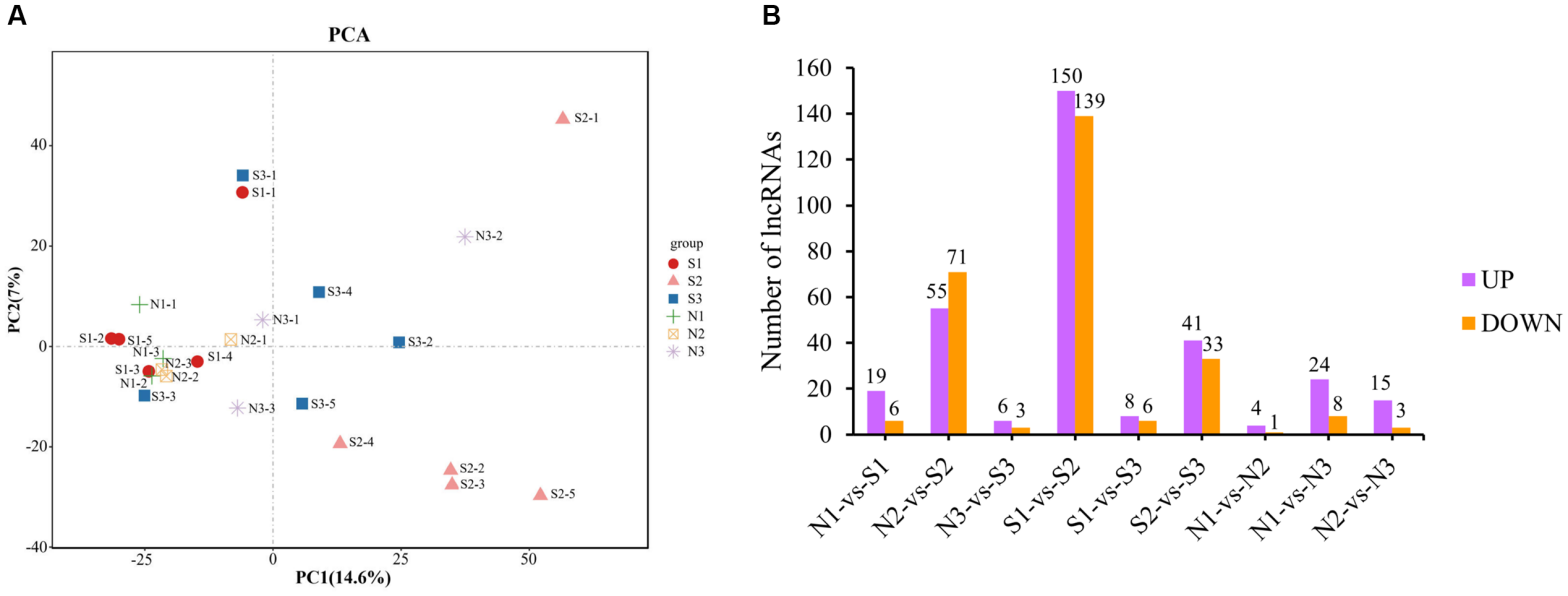
PCA and differential expression analysis of lncRNAs. **(A)** PCA of lncRNAs. Different shapes represent different phases of different groups, for example, the red circle represents the first phase of the S group (S1). **(B)** Statistical diagram of DE lncRNAs. Purple represents up-regulation DE lncRNAs, and orange represents down- regulation DE lncRNAs.

Principal component analysis (PCA) was performed using FPKM values of all lncRNAs to identify the sample clusters and distribution patterns (Figure 2A). For the PCA, the closeness of samples was associated with a stronger positive correlation. For the S group, the samples of S1 were closer to those of S3, whereas those of S2 were away from both because both S1 and S3 were in the anagen phase and S2 was in the telogen phase. Therefore, they were distributed differently in Figure 2A. The three phases in the N group were all anagen, and the difference was that N1 and N2 were in the anagen phase and N3 was in the slow anagen phase, resulting in the samples being close together. There existed outlier samples, such as S1-1 and N3-2, probably because the cluster was affected by the gene’s expression from other tissues in skin samples instead of hair follicles.

#### 3.2.2. Analysis of differential expression lncRNAs

A total of 395 DE lncRNAs were identified. Among them, 313 DE lncRNAs were in the S group (S1-vs-S2, S1-vs-S3, S2-vs-S3), 49 DE lncRNAs in the N group (N1-vs-N2, N1-vs-N3, N2-vs-N3), and 155 DE lncRNAs in the S-vs-N group (S1-vs-N1, S2-vs-N2, S3-vs-N3). The number of DE lncRNAs with the up−/down-regulation in each group is shown in Figure 2B. The S2 group had the highest number of DE lncRNAs compared with others due to the larger difference between S2 in the telogen phase and other phases. However, the number of DE lncRNAs between the other groups was low. These findings are consistent with the results of PCA clustering.

#### 3.2.3. Cluster heatmap and expression pattern analysis of differentially expressed lncRNAs

**Figure 3:**
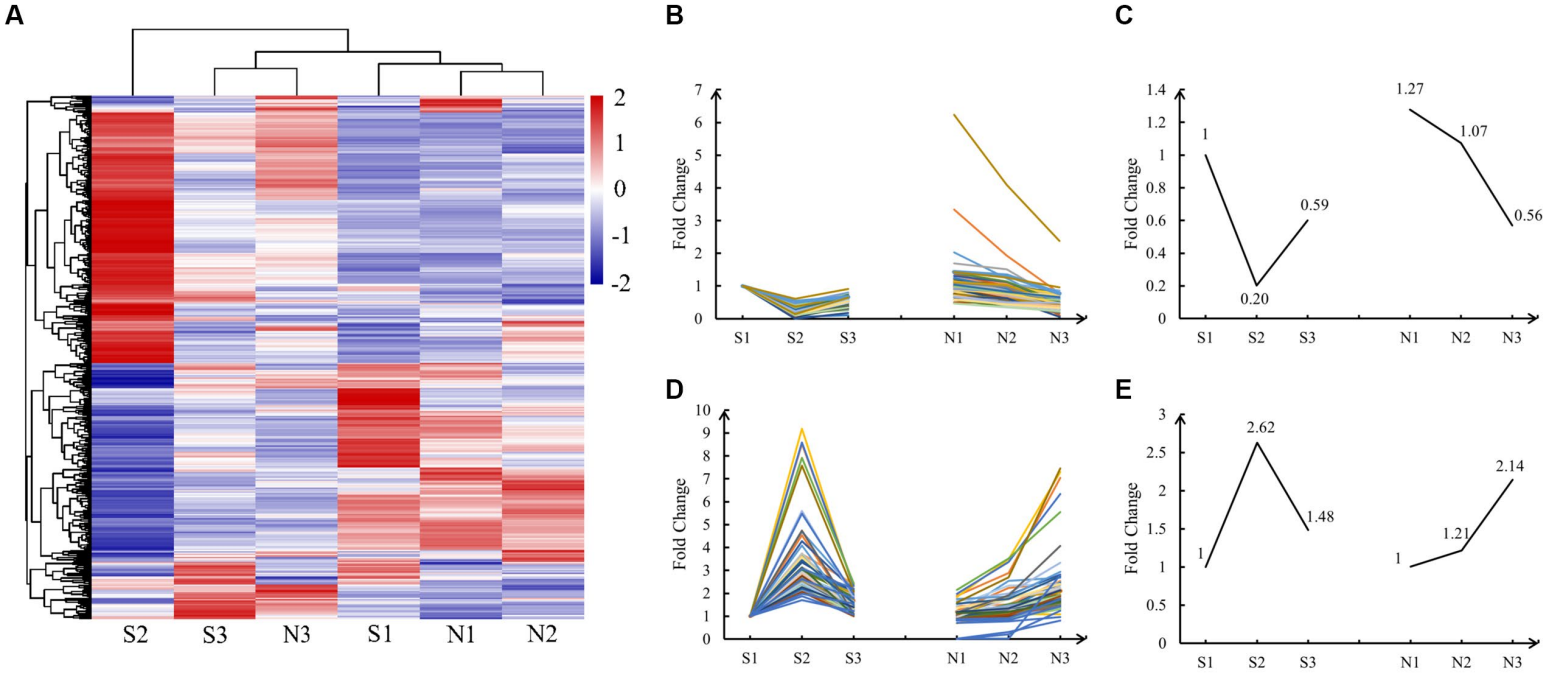
Cluster heatmap and expression pattern analysis of DE lncRNAs. **(A)** Cluster heatmap of normalized expression data for lncRNAs. Positive correlations are marked in red and negative ones in blue, whereas hierarchical cluster illustrates grouping patterns. **(B,D)** The lncRNAs of A and T patterns, and the data have been normalized. **(C,E)** Master pattern plotted using the mean of the normalized FPKM value of DE lncRNAs conforming to the A and T patterns.

Cluster heatmap analysis revealed clustering of similar development stages of hair follicles (Figure 3A). S1, N1, and N2 groups were clustered because they were present in the same active anagen phase in both groups. S3 and N3 groups were clustered together because early anagen and slow anagen phases had similar hair follicle growth states. S2 was a single cluster because the telogen phase was considerably different from other stages.

Cluster heatmap analysis revealed two types of important expression patterns. Pattern one, in which the expression of DE lncRNAs was the highest in the anagen phase (S1, N1, and N2), lowest in S2 (telogen), and middle in the early anagen phase and the slow anagen phase (S3 and N3), was defined as the A (Anagen) pattern. Pattern two, in which the expression of DE lncRNAs was the highest in S2 (telogen), lowest in the anagen phase (S1, N1, and N2), and moderate in the early anagen phase, and in the slow anagen phase (S3 and N3), was defined as the T (Telogen) pattern. We finally identified 57 lncRNAs in the A pattern and 72 lncRNAs in the T pattern, with significant regulatory roles in the anagen and telogen phases, respectively. The FPKM values of these DE lncRNAs were normalized and the expression lines are shown in Figures 3B,D. The mean of all normalized FPKM values in A and T patterns was computed and depicted in Figures 3C,E.

### 3.3. Target gene prediction results of lncRNAs

To investigate the functions of lncRNAs, potential target genes of 129 screened lncRNAs were predicted according to trans-role (|cor| ≥ 0.95), cis-role (upstream and downstream 10 kb), and antisense-role. The 118 (trans-role), 29 (cis-role), and 29 (antisense-role) lncRNAs-target genes pairs were identified by three prediction methods, respectively (Supplementary Table 1). The cor of lncRNAs-target genes pairs in cis-role and antisense-role were further calculated, and 19 pairs with |cor| ≥ 0.8 were left in each. All lncRNAs-target gene pairs screened by three prediction methods were positively correlated, suggesting that the lncRNAs probably positively regulated the corresponding target genes to affect the cyclic growth and development of hair follicles.

### 3.4. Expression pattern analysis of target genes

**Figure 4:**
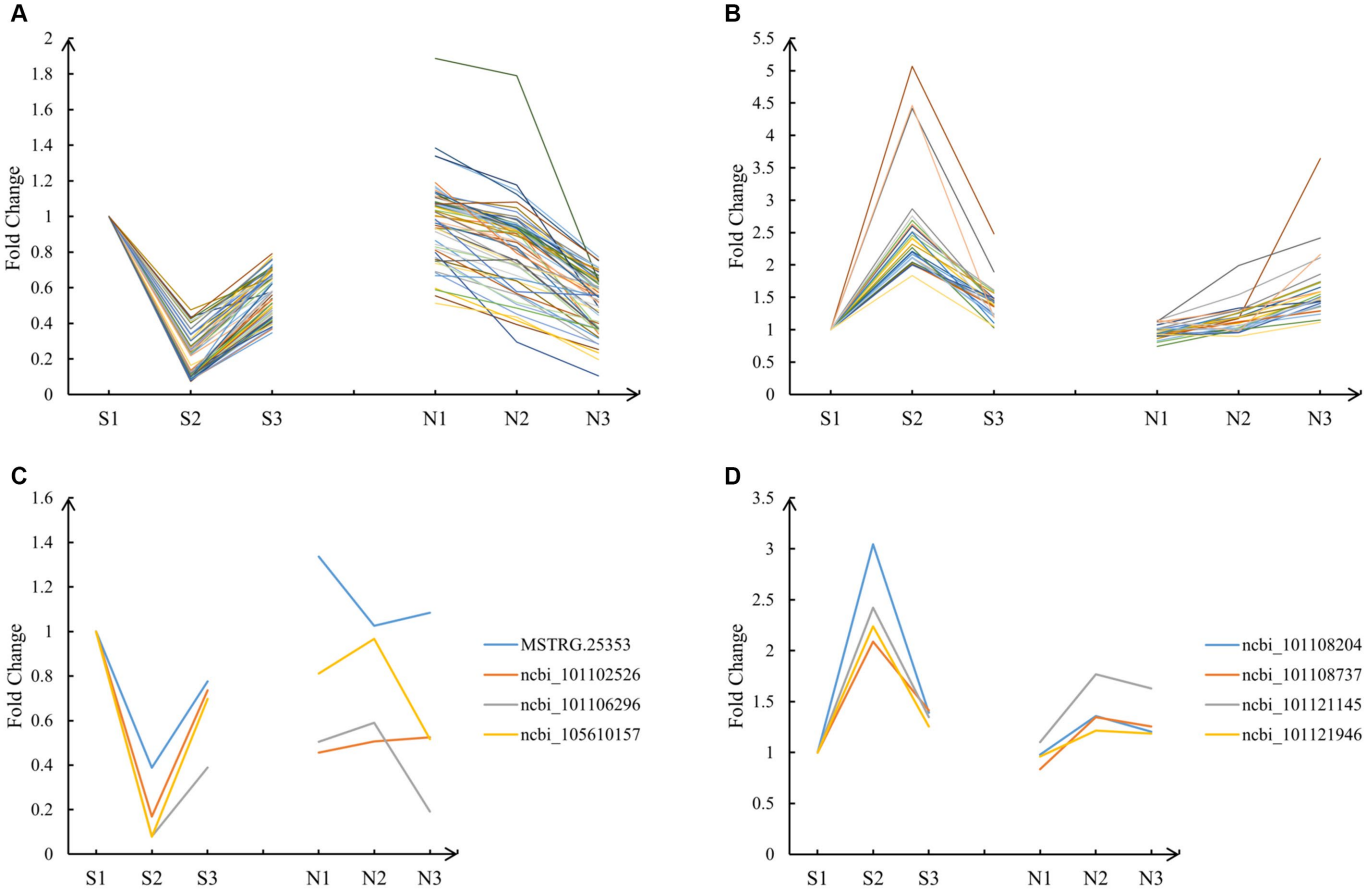
Expression pattern of target genes. **(A)** The 63 DEGs that fitted the A pattern. **(B)** The 28 DEGs that fitted the T pattern. **(C)** The 4 DEGs that did not fit the A pattern. **(D)** The 4 DEGs that did not fit the T pattern.

A prediction of the target genes of lncRNAs resulted in 99 targets (Supplementary Table 2), all of which were differentially expressed genes (DEGs) (FDR < 0.05 and |log2FC|>1 ). Their expression patterns were analyzed. Among them, 63 fitted the A pattern (Figure 4A), 28 fitted the T pattern (Figure 4B), and 8 DEGs did not fit the A and T patterns (Figures 4C,D). The 91 DEGs of A and T patterns were left for subsequent analysis.

### 3.5. GO and KEGG enrichment analysis of target genes

In terms of biological processes, genes were concentrated in cellular process, single-organism process, regulation of biological process, and biological regulation. In terms of molecular functions, genes preferentially corresponded to binding, including protein binding, nucleic acid binding, heterocyclic compound binding, and organic cyclic compound binding-related genes. In terms of cellular components, genes were enriched in organelle, cell and cell part, including genes related to cell structure, connectivity, and communication. The GO enrichment results displayed specific patterns in the growth and development of Dorper sheep hair follicles, thereby contributing to an in-depth understanding of the biological mechanism of hair follicles.

**Figure 5:**
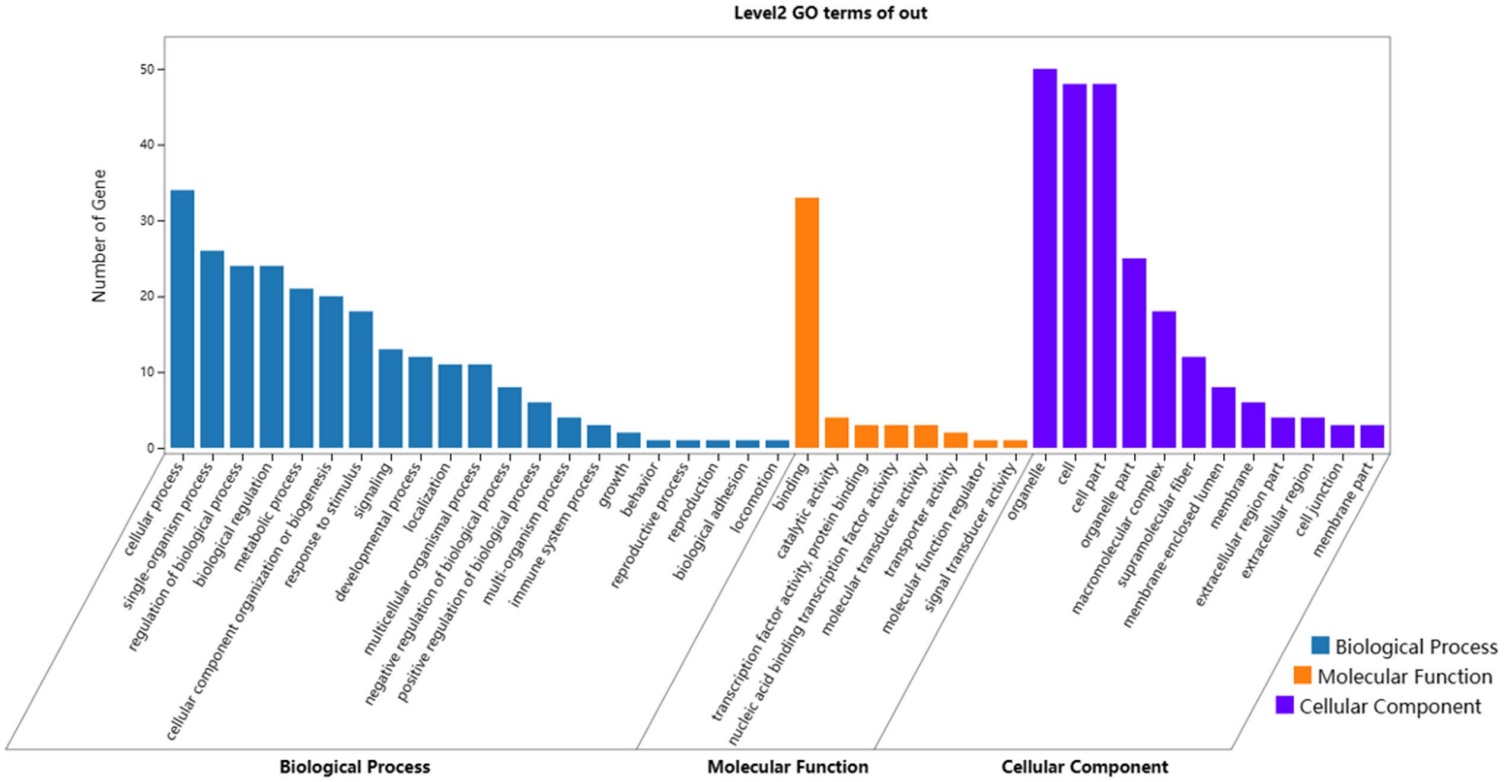
Results of GO enrichment analyses of target genes. Blue, orange, and purple words represent the GO terms in biological processes, molecular functions, and cellular components, respectively. The *x*-axis represents the different GO terms, and the *y*-axis represents the number of genes.

**Figure 6:**
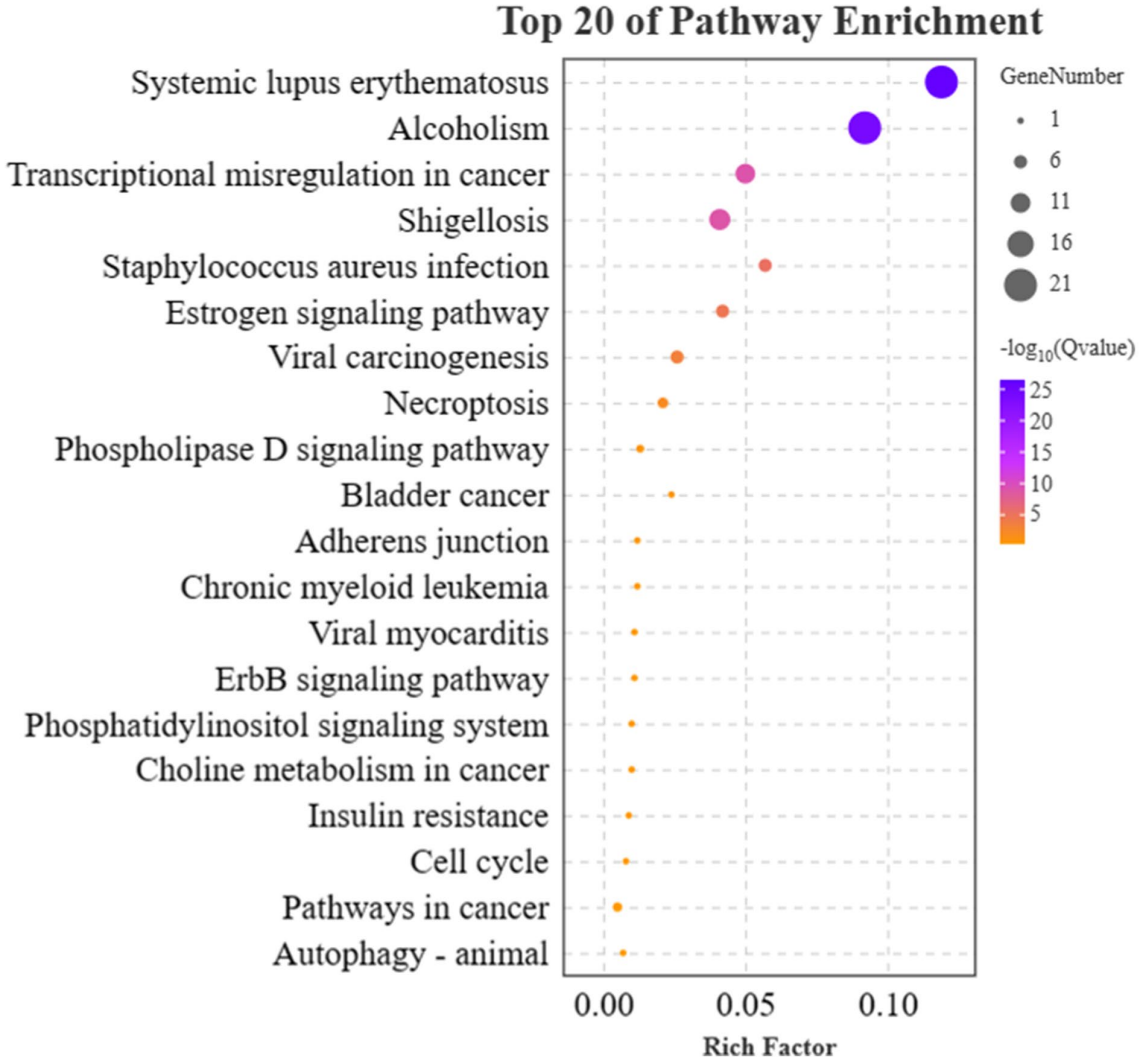
Results of KEGG enrichment analyses of target genes. The *x*-axis and *y*-axis indicate the rich factor and the KEGG pathway names, respectively. The size of the circle represents the number of genes enriched in the pathway. The purple color represents a high enrichment (−log10 of *Q* value).

Next, the KEGG enrichment analysis was performed on 91 DEGs that fitted the A and T patterns. A total of 40 pathways were enriched, and the target genes were significantly enriched in Systemic lupus erythematosus (ko05322), Alcoholism (ko05034), Shigellosis (ko05131), Transcriptional misregulation in cancers (ko05202), *Staphylococcus aureus* infection (ko05150), Estrogen signaling pathway (ko04915), Viral carcinogenesis (ko05203), and Necroptosis (ko04217). The top 20 enriched canonical pathways are shown in Figure 6. Among these 40 pathways, 9 lncRNAs that fitted the A pattern were identified to regulate 12 pathways by targeting 16 corresponding target genes, whereas 8 lncRNAs that fitted the T pattern were identified to regulate 31 pathways by targeting 8 corresponding target genes. The lncRNAs and their corresponding target genes identified in these pathways are summarized in Supplementary Table 3. In addition, the Estrogen signaling pathway (ko04915) and PI3K-Akt signaling pathways (ko04151) were found to be associated with the anagen phase of hair follicles. Similarly, Fc gamma R-mediated phagocytosis (ko04666), Chemokine signaling pathway (ko04062), Bacterial invasion of epithelial cells (ko05100), Cell adhesion molecules (CAMs) (ko04514), and Regulation of actin cytoskeleton (ko04810) are associated with the telogen phase of hair follicles.

### 3.6. Construction of the regulatory network

**Figure 7:**
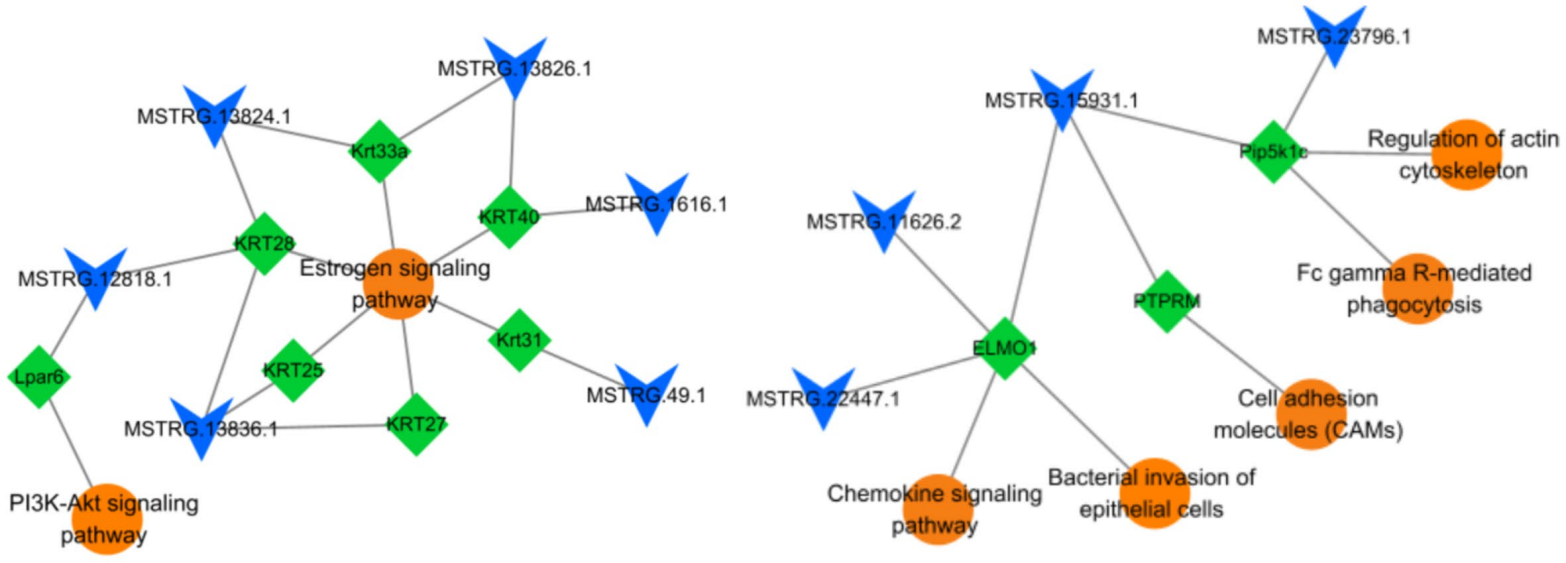
LncRNAs-target genes-pathways association network. A total of 10 lncRNAs, 10 target genes, and 7 signaling pathways are involved in this network. The circle represents pathways, the diamond represents target genes, and V represents lncRNAs.

As shown in Table 3, six candidate lncRNAs (MSTRG.12818.1, MSTRG.13824.1, MSTRG.13836.1, MSTRG.13826.1, MSTRG.1616.1, MSTRG.49.1,) were identified to be involved in the anagen phase by regulating seven corresponding target genes (*KRT28*, *Krt33a*, *KRT40*, *KRT27*, *KRT25*, *Krt31*, *Lpar6*) of the Estrogen (ko04915) and PI3K-Akt signaling pathway (ko04151). Similarly, four candidate lncRNAs (MSTRG.15931.1, MSTRG.22447.1, MSTRG.11626.2, MSTRG.23796.1) were found to play an important role in the telogen phase by regulating three corresponding target genes (*PTPRM*, *ELMO1*, *Pip5k1c*) distributed in five pathways [Fc gamma R-mediated phagocytosis (ko04666), Chemokine signaling pathway (ko04062), Bacterial invasion of epithelial cells (ko05100), Cell adhesion molecules (CAMs) (ko04514), and Regulation of actin cytoskeleton (ko04810)]. Their regulatory network was constructed with Cytoscape (v3.9.1) (Figure 7). In addition, sequencing-related information for 10 important lncRNAs was provided in Supplementary Tables 4, 5.

**Table 3:** Candidate lncRNAs-target genes-pathways correspondence.

Patterns	LncRNAs-target genes	Pathways
A	MSTRG.12818.1/MSTRG.13824.1/MSTRG.13836.1-*KRT28*	Estrogen signaling pathway
	MSTRG.13824.1/MSTRG.13826.1-*Krt33a*	
	MSTRG.13826.1/MSTRG.1616.1-*KRT40*	
	MSTRG.13836.1-*KRT27*	
	MSTRG.13836.1-*KRT25*	
	MSTRG.49.1-*Krt31*	
T	MSTRG.12818.1-*Lpar6*	PI3K-Akt signaling pathway
	MSTRG.15931.1-*PTPRM*	Cell adhesion molecules (CAMs)
	MSTRG.15931.1/MSTRG.22447.1/MSTRG.11626.2-*ELMO1*	Bacterial invasion of epithelial cells
		Chemokine signaling pathway
	MSTRG.15931.1/MSTRG.23796.1-*Pip5k1c*	Fc gamma R-mediated phagocytosis
		Regulation of actin cytoskeleton

### 3.7. qRT-PCR

**Figure 8:**
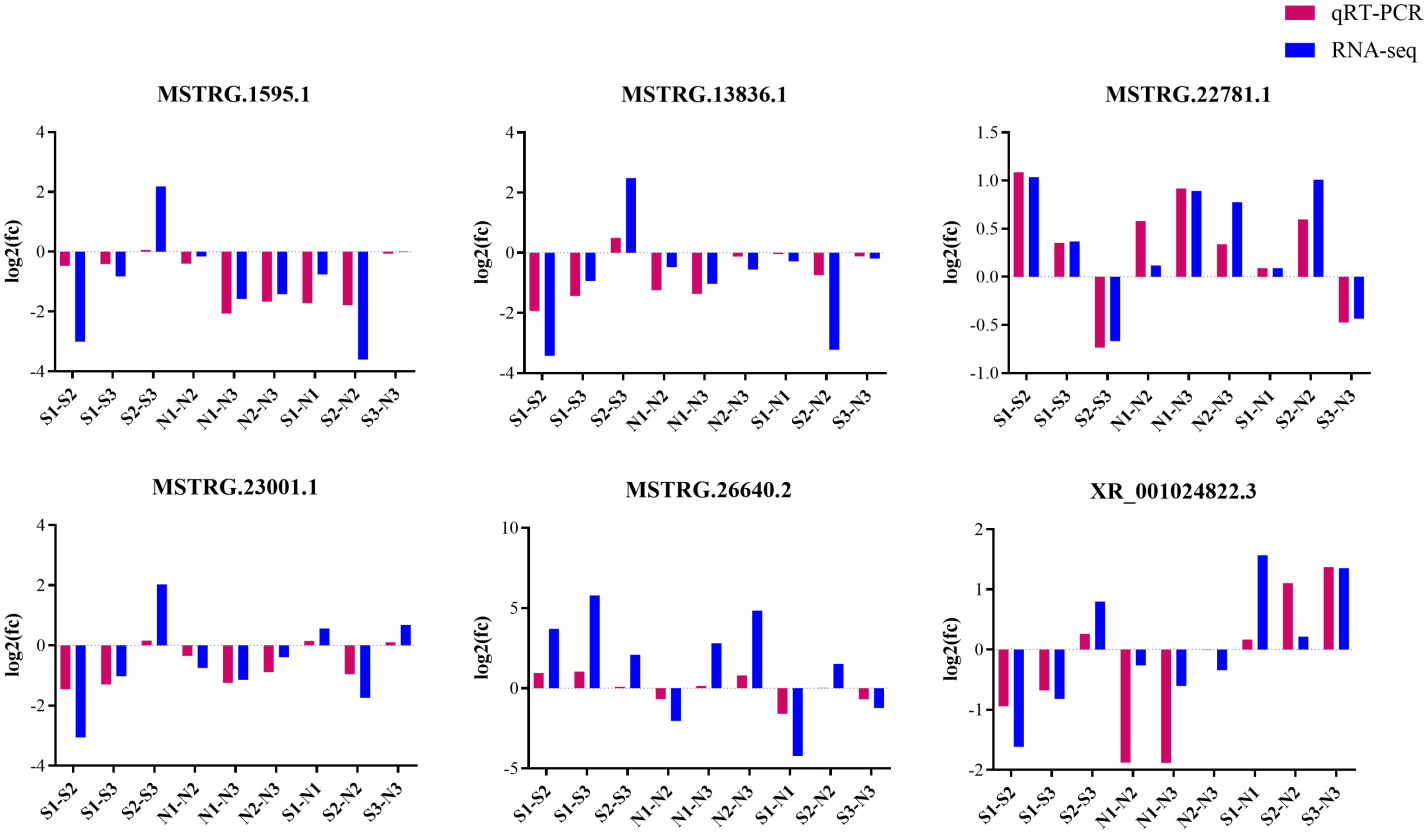
Up−/down-regulation of lncRNAs between qRT-PCR and RNA-seq results.

Six DE lncRNAs were randomly selected for quantitative real-time polymerase chain reaction (qRT-PCR) verification analysis. The verification results were generally consistent with the transcriptome sequencing results, indicating the reliability of our sequencing results (Figure 8).

## 4. Discussion

The hair follicle undergoes three phases of cyclic growth and development, namely, anagen, catagen, and telogen phases (43). LncRNAs have been implicated in the regulatory role of the cyclic growth and development of hair follicles (44). In this study, shedding sheep and non-shedding sheep were used as experimental animals, and lncRNAs affecting the anagen and telogen phases were effectively screened. In addition, their target genes and corresponding pathways enriched to the regulatory network were analyzed.

### 4.1. Validity of lncRNA screening

To study hair follicle cyclic growth and development, the majority of the researchers have used different developmental stages of the same population. For example, Wu et al. selected three 2-year-old female Jiangnan Cashmere goats as experimental animals to screen the candidate lncRNAs, mRNAs, and related pathways affecting the three developmental stages of secondary hair follicles by RNA-seq (45). Similarly, Wang et al. selected five 1-year-old female *Capra hircus* in their comprehensive analysis of genes encoding and non-coding RNAs of the hair follicle cycle, of which three were used to construct and analyze the lncRNA library and two for constructing miRNA library (46). Li et al. screened three Inner Mongolian Cashmere goats for seasonal genes during the cyclic growth and development of hair follicles (47). These studies selected only one population as the experimental animals and screened regulatory molecules such as mRNAs, lncRNAs, and miRNAs related to hair follicle cyclic growth and development. These studies were prone to adulteration of false-positive genes caused by seasonality and other factors. In contrast, we used the shedding population (S1: anagen, S2: telogen, S3: early anagen) of Dorper sheep as experimental animals and the non-shedding population (N1, N2: anagen, N3: slow anagen) as the control. We revealed two lncRNAs expression patterns with high expression in anagen or telogen phases using the cluster heatmap analysis. This experimental design effectively screened the candidate lncRNAs affecting the anagen and telogen phases of hair follicles. The use of a non-shedding population as a control allowed the removal of certain false positives caused by seasonal differential genes, such as MSTRG.11915.2, MSTRG.2866.1, MSTRG.14658.1, and XR_003586196.1 (Supplementary Figure 2A) and other 76 lncRNAs identified in this experiment (Supplementary Table 6). These lncRNAs displayed a consistent expression pattern in both populations. As shown in Figure S1B, 31 lncRNAs exhibited a high-low-high expression pattern at all three time points in both S and N groups. Thus, these DE lncRNAs were affected by the season instead of the cyclic growth and development of hair follicles. We removed these DE lncRNAs with consistent expression patterns. Finally, 129 DE lncRNAs significantly associated with hair follicle anagen and telogen phases were screened for subsequent analysis, which effectively excluded the interference of seasonal factors.

### 4.2. Regulatory role of lncRNAs and corresponding target genes in the cyclic growth and development of hair follicles

The normalized FPKM values of 395 DE lncRNAs were analyzed by clustering heatmap analysis, and 129 DE lncRNAs were analyzed to fit two kinds of expression patterns (A and T patterns), among which 57 lncRNAs were found to play regulatory roles in the anagen (A pattern) and 72 lncRNAs in telogen (T pattern) phases. The target genes of these lncRNAs were predicted using three methods (co-localization, co-expression, and antisense effects), combined with GO and KEGG functional analyses, to explore the regulatory functions of candidate lncRNAs in anagen and telogen phases of hair follicles.

Finally, six lncRNAs (MSTRG.12818.1, MSTRG.13824.1, MSTRG.13836.1, MSTRG.13826.1, MSTRG.1616.1, MSTRG.49.1) as potential candidates were screened to play regulatory functions in the anagen phase by targeting seven genes (*KRT28*, *Krt33a*, *KRT40*, *KRT27*, *KRT25*, *Krt31*, *Lpar6*) of the Estrogen (ko04915) and PI3K-Akt signaling pathways (ko04151) (Table 3). Among them, six lncRNAs functioned in the Estrogen signaling pathway (ko04915) by regulating six keratins (*KRT28*, *Krt33a*, *KRT40*, *KRT27*, *KRT25*, *Krt31*). Wang (48) and Wang et al. (49) reported the Estrogen signaling pathway (ko04915) as a crucial pathway during the anagen phase of hair follicles and that its activation promoted the expression of keratin. Keratin is an important component of hairs and Yu (50), Morgan (51), and Fukuyama et al. (52) reported the abundant expression of these keratins in hair follicle growth and development. The expression of the keratins is a marker of hair follicle growth and development (53). Among them, MSTRG.13836.1-*KRT28* was screened by both trans-role and antisense-role analyses and was used for further study. Wang et al. identified crucial pathways and genes associated with hair follicle cyclic growth and development in cashmere goats and reported that genes associated with the anagen phase were enriched in the PI3K-Akt signaling pathway (ko04151) (49). The PI3K-Akt signaling pathway (ko04151) is essential for maintaining and restoring hair induction in dermal papilla cells (54) and promoting proliferation and inhibiting apoptosis in dermal papilla cells (55). *Lpar6* was detected to be enriched in the PI3K-Akt signaling pathway (ko04151) in this study. Therefore, it was hypothesized that MSTRG.12818.1 plays a regulatory role in the PI3K-Akt signaling pathway (ko04151) by targeting *Lpar6* to affect the anagen phase of hair follicles.

We screened four lncRNAs (MSTRG.15931.1, MSTRG.22447.1, MSTRG.11626.2, MSTRG.23796.1) as potential candidates and found them to be implicated in the telogen phase of hair follicles by regulating three corresponding target genes (*PTPRM*, *ELMO1*, *Pip5k1c*) distributed in five pathways (Table 3). Four pathways, namely, Fc gamma R-mediated phagocytosis (ko04666), Chemokine signaling pathway (ko04062), Bacterial invasion of epithelial cells (ko05100), and Cell adhesion molecules (CAMs) (ko04514) were found to be associated with the telogen phase of hair follicles (47). Among them, Fc gamma R-mediated phagocytosis (ko04666) was associated with downstream hair follicle atrophy during the telogen phase (48). Nagao et al. demonstrated that chemokines can induce immune cell migration following the entry of hair follicles into the catagen phase, thus further promoting the apoptosis of hair follicle cells (56). Interestingly, lncRNA MSTRG.15931.1 was predicted to target *PTPRM*, *ELMO1*, and *Pip5k1c*. Among them, *PTPRM* has been reported to regulate intercellular communication in keratin-forming cells (57), and *ELMO1* has been demonstrated to play an unexpected role in clearing apoptotic cells from hair follicles and maintaining homeostasis (58). Both *ELMO1* (59) and *Pip5k1c* (60) were implicated in the remodeling of the actin cytoskeleton via the fifth pathway, i.e., the Regulation of the actin cytoskeleton (ko04810). Therefore, the specific regulatory function of lncRNA MSTRG.15931.1 in the telogen phase of hair follicles will be the focus of our further research.

## 5. Conclusion

In this study, 129 lncRNAs were screened for their expression patterns to elucidate their regulatory roles in hair follicle growth and development during the anagen and telogen phases in Dorper sheep. In total, 10 candidate lncRNAs (MSTRG.12818.1, MSTRG.13824.1, MSTRG.13836.1, MSTRG.13826.1, MSTRG.1616.1, MSTRG.49.1, MSTRG.15931.1, MSTRG.22447.1, MSTRG.11626.2, MSTRG.23796.1) were identified by predicting target genes and through functional enrichment analysis, among which six lncRNAs (MSTRG.12818.1, MSTRG.13824.1, MSTRG.13836.1, MSTRG.13826.1, MSTRG.1616.1, MSTRG.49.1) were found to play an important regulatory role in the anagen phase and four lncRNAs (MSTRG.15931.1, MSTRG.22447.1, MSTRG.11626.2, MSTRG.23796.1) in the telogen phase.

## Data availability statement

The datasets presented in this study can be found in online repositories. The names of the repository/repositories and accession number(s) can be found at: https://www.ncbi.nlm.nih.gov/bioproject; PRJNA963059.

## Ethics statement

The animal study was reviewed and approved by Experimental Animal Welfare and Ethics Committee of Ningxia University.

## Author contributions

XL and HS contributed to the conception and design of the study. HS organized the database, performed statistical analysis, and wrote the first draft of the manuscript. KM, YFW, YYW, and XY provided the usage of some of the software. XL completed the revision of the manuscript. All authors contributed to the manuscript revision, read, and approved the submitted version.

## Funding

This study was financially supported by the National Natural Science Foundation of China “Screening of key molecules and construction of regulatory network about sheep shedding trait” (No. 31960650).

## Conflict of interest

The authors declare that the research was conducted in the absence of any commercial or financial relationships that could be construed as a potential conflict of interest.

## Publisher’s note

All claims expressed in this article are solely those of the authors and do not necessarily represent those of their affiliated organizations, or those of the publisher, the editors and the reviewers. Any product that may be evaluated in this article, or claim that may be made by its manufacturer, is not guaranteed or endorsed by the publisher.

## Footnotes

^1^http://www.r-project.org/

^2^www.omicshare.com/tools

## Supplementary material

SUPPLEMENTARY FIGURE S1 Flow chart of lncRNA analysis.

SUPPLEMENTARY FIGURE S2 Expression patterns of DE lncRNAs (n=80) influenced by seasonal factors.

SUPPLEMENTARY TABLE S1 The lncRNA-target gene pairs screened by three target gene prediction methods.

SUPPLEMENTARY TABLE S2 The 99 target genes obtained by lncRNA target gene prediction.

SUPPLEMENTARY TABLE S3 The lncRNAs and their corresponding target genes identified in 40 pathways.

SUPPLEMENTARY TABLE S4 Sequencing information of 10 candidate lncRNAs.

SUPPLEMENTARY TABLE S5 Sequences of 10 candidate lncRNAs.

SUPPLEMENTARY TABLE S6 The DE lncRNAs (n=80) influenced by seasonal factors.

## References

[1] Liu G Liu R Tang X Cao J Zhao S Yu M. Expression profiling reveals genes involved in the regulation of wool follicle bulb regression and regeneration in sheep. Int J Mol Sci. (2015) 16:9152–66. doi: 10.3390/ijms16059152, PMID: 25915029 PMC4463583

[2] Suen W-J Li S-T Yang L-T. Hes1 regulates anagen initiation and hair follicle regeneration through modulation of hedgehog signaling. Stem Cells. (2020) 38:301–14. doi: 10.1002/stem.3117, PMID: 31721388 PMC7027765

[3] Lin X Zhu L He J. Morphogenesis, growth cycle and molecular regulation of hair follicles. Front Cell Dev Biol. (2022) 10:10. doi: 10.3389/fcell.2022.899095 PMC9133560 35646909

[4] Zhao B Li J Zhang X Dai Y Yang N Bao Z. Exosomal miRNA-181a-5p from the cells of the hair follicle dermal papilla promotes the hair follicle growth and development via the Wnt/β-catenin signaling pathway. Int J Biol Macromol. (2022) 207:110–20. doi: 10.1016/j.ijbiomac.2022.02.177, PMID: 35248611

[5] Öztürk ÖA Pakula H Chmielowiec J Qi J Stein S Lan L. Gab1 and Mapk signaling are essential in the hair cycle and hair follicle stem cell quiescence. Cell Rep. (2015) 13:561–72. doi: 10.1016/j.celrep.2015.09.015 26456821

[6] Telerman SB Rognoni E Sequeira I Pisco AO Lichtenberger BM Culley OJ. Dermal Blimp1 acts downstream of epidermal TGFβ and Wnt/β-catenin to regulate hair follicle formation and growth. J Investig Dermatol. (2017) 137:2270–81. doi: 10.1016/j.jid.2017.06.015, PMID: 28668474 PMC5646946

[7] Calvo-Sánchez MI Fernández-Martos S Carrasco E Moreno-Bueno G Bernabéu C Quintanilla M. A role for the Tgf-β/bmp co-receptor Endoglin in the molecular oscillator that regulates the hair follicle cycle. J Mol Cell Biol. (2019) 11:39–52. doi: 10.1093/jmcb/mjy051, PMID: 30239775 PMC6359924

[8] Pujades C Kamaid A Alsina B Giraldez F. BMP-signaling regulates the generation of hair-cells. Dev Biol. (2006) 292:55–67. doi: 10.1016/j.ydbio.2006.01.001, PMID: 16458882

[9] Kan L Liu Y McGuire TL Bonaguidi MA Kessler JA. Inhibition of BMP signaling in P-cadherin positive hair progenitor cells leads to trichofolliculoma-like hair follicle neoplasias. J Biomed Sci. (2011) 18:92–12. doi: 10.1186/1423-0127-18-92 22168923 PMC3262035

[10] Nissimov JN Das Chaudhuri AB. Hair curvature: A natural dialectic and review. Biol Rev. (2014) 89:723–66. doi: 10.1111/brv.12081, PMID: 24617997

[11] Ohuchi H Tao H Ohata K Itoh N Kato S Noji S. Fibroblast growth factor 10 is required for proper development of the mouse whiskers. Biochem Biophys Res Commun. (2003) 302:562–7. doi: 10.1016/S0006-291X(03)00183-9, PMID: 12615071

[12] Mimeault M Batra SK. Hypoxia-inducing factors as master regulators of stemness properties and altered metabolism of cancer-and metastasis-initiating cells. J Cell Mol Med. (2013) 17:30–54. doi: 10.1111/jcmm.12004, PMID: 23301832 PMC3560853

[13] Driskell RR Clavel C Rendl M Watt FM. Hair follicle dermal papilla cells at a glance. J Cell Sci. (2011) 124:1179–82. doi: 10.1242/jcs.082446, PMID: 21444748 PMC3115771

[14] Christiano AM. Hair follicle epithelial stem cells get their sox on. Cell Stem Cell. (2008) 3:3–4. doi: 10.1016/j.stem.2008.06.014, PMID: 18593550

[15] Tomann P Paus R Millar SE Scheidereit C Schmidt-Ullrich R. Lhx2 is a direct NF-κB target gene that promotes primary hair follicle placode down-growth. Development. (2016) 143:1512–22. doi: 10.1242/dev.130898, PMID: 26952977 PMC6514410

[16] Lei M Guo H Qiu W Lai X Yang T Widelitz RB. Modulating hair follicle size with W nt10b/DKK 1 during hair regeneration. Exp Dermatol. (2014) 23:407–13. doi: 10.1111/exd.12416, PMID: 24750467 PMC4383245

[17] Yan H Jin M Li Y Gao Y Ding Q Wang X. miR-1 regulates differentiation and proliferation of goat hair follicle stem cells by targeting IGF1R and LEF1 genes. DNA Cell Biol. (2022) 41:190–201. doi: 10.1089/dna.2021.0288, PMID: 35007429

[18] Botchkarev VA Botchkareva NV Nakamura M Huber O Funa K Lauster R. Noggin is required for induction of the hair follicle growth phase in postnatal skin. FASEB J. (2001) 15:2205–14. doi: 10.1096/fj.01-0207com, PMID: 11641247

[19] Sha K Chen M Liu F Xu S Wang B Peng Q. Platelet factor 4 inhibits human hair follicle growth and promotes androgen receptor expression in human dermal papilla cells. PeerJ. (2020) 8:e9867. doi: 10.7717/peerj.9867, PMID: 32953277 PMC7476492

[20] Zhao J Lin H Wang L Guo K Jing R Li X. Suppression of FGF5 and FGF18 expression by cholesterol-modified siRNAs promotes hair growth in mice. Front Pharmacol. (2021) 12:666860. doi: 10.3389/fphar.2021.666860, PMID: 34305588 PMC8293299

[21] Yue Y Guo T Yuan C Liu J Guo J Feng R. Integrated analysis of the roles of long noncoding RNA and coding RNA expression in sheep (*Ovis aries*) skin during initiation of secondary hair follicle. PLoS One. (2016) 11:e0156890. doi: 10.1371/journal.pone.0156890, PMID: 27276011 PMC4898689

[22] Guo Y. Screening of specific long non-coding RNAs regulation periodic growth of cashmere. Master’s thesis. Shan Xi: Northwest A&F University (2015).

[23] Si Y Bai J Wu J Li Q Mo Y Fang R. LncRNA PlncRNA-1 regulates proliferation and differentiation of hair follicle stem cells through TGF-β1-mediated Wnt/β-catenin signal pathway. Mol Med Rep. (2018) 17:1191–7. doi: 10.3892/mmr.2017.7944, PMID: 29115537

[24] Chen S Zhou Y Chen Y Gu J. fastp: an ultra-fast all-in-one FASTQ preprocessor. Bioinformatics. (2018) 34:i884–90. doi: 10.1093/bioinformatics/bty560, PMID: 30423086 PMC6129281

[25] Kim D Langmead B Salzberg SL. HISAT: a fast spliced aligner with low memory requirements. Nat Methods. (2015) 12:357–60. doi: 10.1038/nmeth.3317, PMID: 25751142 PMC4655817

[26] Pertea M Pertea GM Antonescu CM Chang T-C Mendell JT Salzberg SL. StringTie enables improved reconstruction of a transcriptome from RNA-seq reads. Nat Biotechnol. (2015) 33:290–5. doi: 10.1038/nbt.3122, PMID: 25690850 PMC4643835

[27] Kang Y-J Yang D-C Kong L Hou M Meng Y-Q Wei L. CPC2: a fast and accurate coding potential calculator based on sequence intrinsic features. Nucleic Acids Res. (2017) 45:W12–6. doi: 10.1093/nar/gkx428, PMID: 28521017 PMC5793834

[28] Sun L Luo H Bu D Zhao G Yu K Zhang C. Utilizing sequence intrinsic composition to classify protein-coding and long non-coding transcripts. Nucleic Acids Res. (2013) 41:e166. doi: 10.1093/nar/gkt646 23892401 PMC3783192

[29] Nawrocki EP Eddy SR. Infernal 1.1: 100-fold faster RNA homology searches. Bioinformatics. (2013) 29:2933–5. doi: 10.1093/bioinformatics/btt509, PMID: 24008419 PMC3810854

[30] Cole T Adam R Loyal G Geo P Daehwan K KD R. Differential gene and transcript expression analysis of RNA-seq experiments with TopHat and cufflinks. Nat Protoc. (2012) 7:562–78. doi: 10.1038/nprot.2012.016 22383036 PMC3334321

[31] Li B Dewey CN. RSEM: accurate transcript quantification from RNA-Seq data with or without a reference genome. BMC Bioinformatics. (2011) 12:1–16. doi: 10.1186/1471-2105-12-323 21816040 PMC3163565

[32] Love MI Huber W Anders S. Moderated estimation of fold change and dispersion for RNA-seq data with DESeq2. Genome Biol. (2014) 15:1–21. doi: 10.1186/s13059-014-0550-8 PMC4302049 25516281

[33] Wang Y. Screening of genes related to shedding traits in Dupo sheep and functional analysis of differential genes. Master’s thesis. Ning Xia: Ning Xia University (2022).

[34] Sònia G Manel E. Cis-acting noncoding RNAs: friends and foes. Nat Struct Mol Biol. (2012) 19:1068–75. doi: 10.1038/nsmb.2428 23132386

[35] Chunmei D Kun W Yiguang Z Xuemei N Ruipeng C Suyu Q. Supplementation with Milk-derived extracellular vesicles shapes the gut microbiota and regulates the transcriptomic landscape in experimental colitis. Nutrients. (2022) 14:1808. doi: 10.3390/nu14091808 35565775 PMC9104790

[36] Liang G Kunwei S Ni Y Min J. Comprehensive transcriptomic analysis reveals dysregulated competing endogenous RNA network in endocrine resistant breast Cancer cells. Front Oncol. (2020) 10:600487. doi: 10.3389/fonc.2020.600487 33324567 PMC7723334

[37] Villegas V Zaphiropoulos P. Neighboring gene regulation by antisense long non-coding RNAs. IJMS. (2015) 16:3251–66. doi: 10.3390/ijms16023251 25654223 PMC4346893

[38] Brown T Howe F Murray S Wouters M Lorenz P Seward E. Antisense transcription-dependent chromatin signature modulates sense transcript dynamics. Mol Syst Biol. (2018) 14:e8007. doi: 10.15252/msb.20178007, PMID: 29440389 PMC5810148

[39] Tafer H Hofacker IL. RNAplex: a fast tool for RNA–RNA interaction search. Bioinformatics. (2008) 24:2657–63. doi: 10.1093/bioinformatics/btn193, PMID: 18434344

[40] Su G Morris JH Demchak B Bader GD. Biological network exploration with Cytoscape 3. Curr Protoc Bioinformatics. (2014) 47:1–8. doi: 10.1002/0471250953.bi0813s47 PMC4174321 25199793

[41] Lalitha S. Primer premier 5. Biotech Softw Internet Rep. (2000) 1:270–2. doi: 10.1089/152791600459894

[42] Livak KJ Schmittgen TD. Analysis of relative gene expression data using real-time quantitative PCR and the 2− ΔΔCT method. Methods. (2001) 25:402–8. doi: 10.1006/meth.2001.1262 11846609

[43] Plowman JE Harland DP. The follicle cycle in brief. The hair fibre: proteins. Structure and Development. (2018) 1054:15–7. doi: 10.1007/978-981-10-8195-8_2 29797264

[44] Zhou G Kang D Ma S Wang X Gao Y Yang Y. Integrative analysis reveals ncRNA-mediated molecular regulatory network driving secondary hair follicle regression in cashmere goats. BMC Genomics. (2018) 19:1–16. doi: 10.1186/s12864-018-4603-3 29587631 PMC5870523

[45] Wu C Qin C Fu X Huang X Tian K. Integrated analysis of lncRNAs and mRNAs by RNA-Seq in secondary hair follicle development and cycling (anagen, catagen and telogen) of Jiangnan cashmere goat (*Capra hircus*). BMC Vet Res. (2022) 18:167. doi: 10.1186/s12917-022-03253-0, PMID: 35524260 PMC9074311

[46] Wang S Ge W Luo Z Guo Y Jiao B Qu L. Integrated analysis of coding genes and non-coding RNAs during hair follicle cycle of cashmere goat (*Capra hircus*). BMC Genomics. (2017) 18:1–13. doi: 10.1186/s12864-017-4145-0 29020916 PMC5637055

[47] Li C Feng C Ma G Fu S Chen M Zhang W. Time-course RNA-seq analysis reveals stage-specific and melatonin-triggered gene expression patterns during the hair follicle growth cycle in *Capra hircus*. BMC Genomics. (2022) 23:1–16. doi: 10.1186/s12864-022-08331-z 35172715 PMC8848980

[48] Wang Y. Screening and validation of genes associated with shedding traits in Dorper sheep. Master’s thesis. Ning Xia: Ning Xia University (2022).

[49] Wang J Sui J Mao C Li X Chen X Liang C. Identification of key pathways and genes related to the development of hair follicle cycle in cashmere goats. Genes. (2021) 12:180. doi: 10.3390/genes12020180, PMID: 33513983 PMC7911279

[50] Yu Z Wildermoth JE Wallace OA Gordon SW Maqbool NJ Maclean PH. Annotation of sheep keratin intermediate filament genes and their patterns of expression. Exp Dermatol. (2011) 20:582–8. doi: 10.1111/j.1600-0625.2011.01274.x, PMID: 21554405

[51] Morgan H Benketah A Olivero C Rees E Ziaj S Mukhtar A. Hair follicle differentiation-specific keratin expression in human basal cell carcinoma. Clin Exp Dermatol. (2020) 45:417–25. doi: 10.1111/ced.14113, PMID: 31580512

[52] Fukuyama M Tsukashima A Kimishima M Yamazaki Y Okano H Ohyama M. Human iPS cell-derived cell aggregates exhibited dermal papilla cell properties in in vitro three-dimensional assemblage mimicking hair follicle structures. Front Cell Dev Biol. (2042) 9:590333. doi: 10.3389/fcell.2021.590333 PMC8365839 34409023

[53] Gao WZ Xue HL Yang JC. Proteomics analysis of the secondary hair follicle cycle in Liaoning cashmere goat. Small Rumin Res. (2021) 201:106408. doi: 10.1016/j.smallrumres.2021.106408

[54] Yamane M Seo J Zhou Y Asaba T Tu S Nanmo A. Effects of the PI3K/Akt signaling pathway on the hair inductivity of human dermal papilla cells in hair beads. J Biosci Bioeng. (2022) 134:55–61. doi: 10.1016/j.jbiosc.2022.03.010, PMID: 35431119

[55] Zong B. A study on the effects of melatonin on the proliferation and apoptosis of dermal papilla cells in primary hair follicle of cashmere goats. Master’s thesis. Shan Xi: Northwest A&F University (2021).

[56] Nagao K Kobayashi T Moro K Ohyama M Adachi T Kitashima DY. Stress-induced production of chemokines by hair follicles regulates the trafficking of dendritic cells in skin. Nat Immunol. (2012) 13:744–52. doi: 10.1038/ni.2353, PMID: 22729248 PMC4115277

[57] Peng H Park JK Katsnelson J Kaplan N Yang W Getsios S. microRNA-103/107 family regulates multiple epithelial stem cell characteristics. Stem Cells. (2015) 33:1642–56. doi: 10.1002/stem.1962, PMID: 25639731 PMC4409488

[58] Elliott MR Zheng S Park D Woodson RI Reardon MA Juncadella IJ. Unexpected requirement for ELMO1 in clearance of apoptotic germ cells in vivo. Nature. (2010) 467:333–7. doi: 10.1038/nature09356, PMID: 20844538 PMC3773546

[59] Grimsley CM Lu M Haney LB Kinchen JM Ravichandran KS. Characterization of a novel interaction between ELMO1 and ERM proteins. J Biol Chem. (2006) 281:5928–37. doi: 10.1074/jbc.M510647200, PMID: 16377631

[60] Ewies AA Elshafie M Li J Stanley A Thompson J Styles J. Changes in transcription profile and cytoskeleton morphology in pelvic ligament fibroblasts in response to stretch: the effects of estradiol and levormeloxifene. Mol Hum Reprod. (2008) 14:127–35. doi: 10.1093/molehr/gam090, PMID: 18184756

