# Supplementary figures and images for: LncRNAs regulate the cyclic growth and development of hair follicles in Dorper sheep
Source: Front Vet Sci. 2023 Jul 31;10:1186294. doi: 10.3389/fvets.2023.1186294 (PMC10423938; doi:10.3389/fvets.2023.1186294)

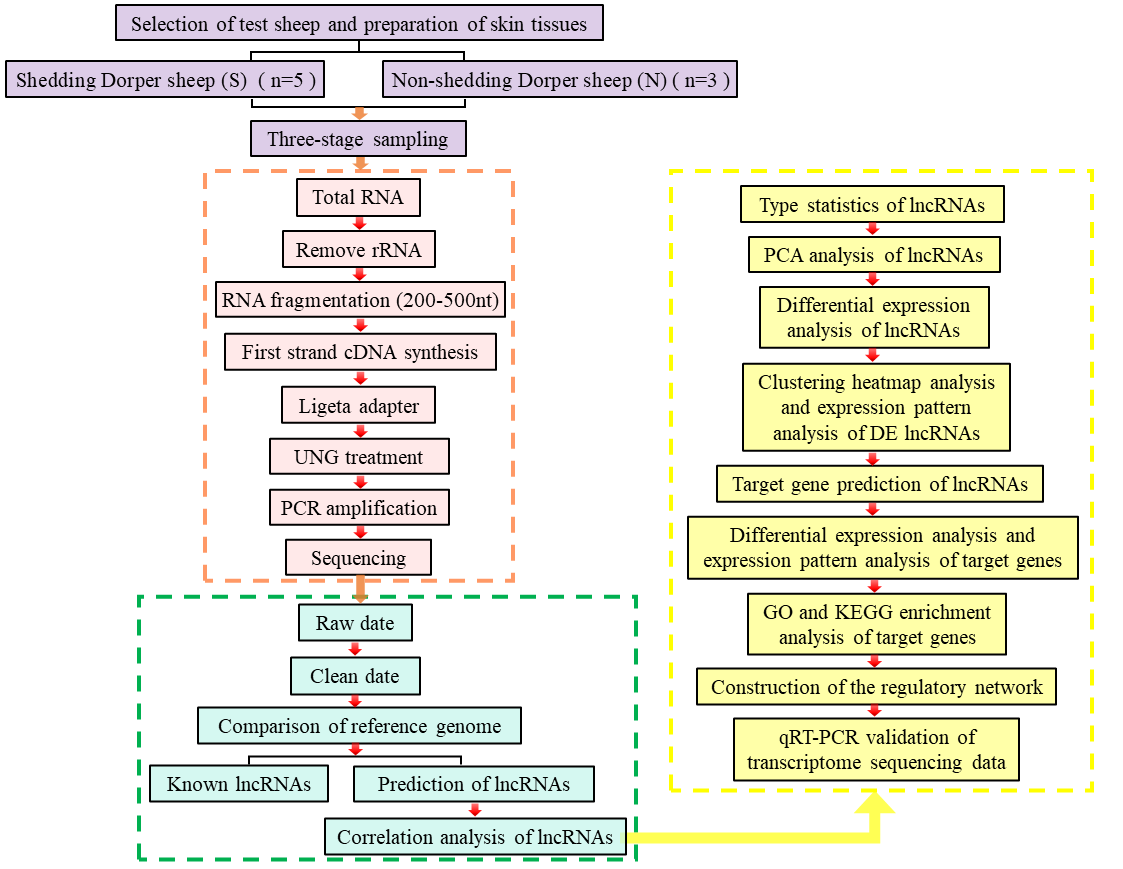

Supplement: SUPPLEMENTARY FIGURE S1 — Flow chart of lncRNA analysis. [file Image_1.TIF]

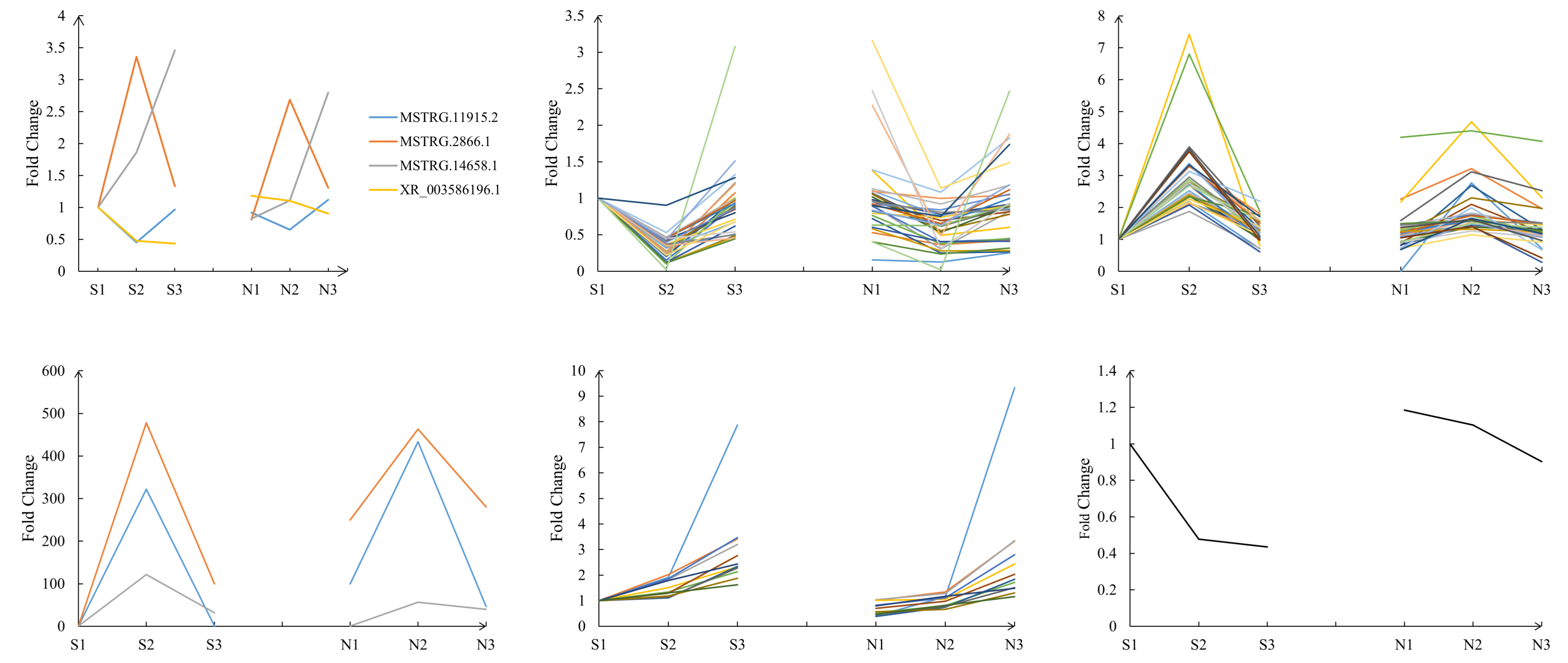

Supplement: SUPPLEMENTARY FIGURE S2 — Expression patterns of DE lncRNAs (n=80) influenced by seasonal factors. [file Image_2.TIF]
